# Older Adults Accessing HIV Care and Treatment and Adherence in the IeDEA Central Africa Cohort

**DOI:** 10.1155/2012/725713

**Published:** 2012-02-16

**Authors:** Jamie Newman, Jeniffer Iriondo-Perez, Jennifer Hemingway-Foday, Anna Freeman, Wilfred Akam, Ashu Balimba, Lucien Kalenga, Marcel Mbaya, Brigitte Mfangam Molu, Henri Mukumbi, Théodore Niyongabo, Joseph Atibu, Innocent Azinyue, Modeste Kiumbu

**Affiliations:** ^1^Statistics and Epidemiology, RTI International, Research Triangle Park, NC 27709-2194, USA; ^2^Limbe Provincial Hospital, Limbe, Cameroon; ^3^Hôpital Militaire de Yaoundé, Yaoundé, Cameroon; ^4^AMO-Congo, Lubumbashi, Democratic Republic of Congo; ^5^AMO-Congo, Matadi, Democratic Republic of Congo; ^6^Hôpital Général de Yaoundé, Yaoundé, Cameroon; ^7^AMO-Congo, Kinshasa, Democratic Republic of Congo; ^8^Centre Hospitalo-Universitaire de Kamenge, Bujumbura, Burundi; ^9^Ecole de Santé Publique, Kinshasa, Democratic Republic of Congo; ^10^Vindata Solutions, Yaoundé, Cameroon

## Abstract

*Background*. Very little is known about older adults accessing HIV care in sub-Saharan Africa. *Materials and Methods*. Data were obtained from 18,839 HIV-positive adults at 10 treatment programs in Burundi, Cameroon, and the Democratic Republic of Congo. We compared characteristics of those aged 50+ with those aged 18–49 using chi-square tests. Logistic regression was used to determine if age was associated with medication adherence. *Results*. 15% of adults were 50+ years. Those aged 50+ were more evenly distributed between women and men (56% versus 44%) as compared to those aged 18–49 (71% versus 29%) and were more likely to be hypertensive (8% versus 3%) (*P* < 0.05). Those aged 50+ were more likely to be adherent to their medications than those aged 18–49 (*P* < 0.001). Adults who were not heavy drinkers reported better adherence as compared to those who reported drinking three or more alcoholic beverages per day (*P* < 0.001). *Conclusions*. Older adults differed from their younger counterparts in terms of medication adherence, sociodemographic, behavioral, and clinical characteristics.

## 1. Introduction

2.8 million people living with HIV worldwide are over the age of 50 [1]. In the USA, 24% of all people living with HIV are older than 50 [2]. In sub-Saharan Africa, more than 14% of adults with HIV are 50 years or older, and this population is growing [3]. Perceived risk of contracting HIV among older adults is low [4] despite physiological changes associated with aging which place older adults at increased risk of contracting HIV [5, 6]. HIV disease progresses more rapidly among older adults than among their younger counterparts, and mortality among older adults is higher after developing an AIDS-defining illness [7, 8].

Older adults are more likely to be diagnosed at late stage of HIV disease progression than their younger counterparts [8, 9]. This may be due, in part, to low perceived susceptibility of HIV among older adults [10] as well as their healthcare providers [11]. Orel et al. [12] evaluated state departments of public health in the USA and concluded that there is a dearth of HIV/AIDS risk-reduction materials targeting older adults. A study conducted in eight sub-Saharan African countries found that older adults had lower levels of knowledge about HIV, and, among older adults, women had the lowest levels of HIV-related knowledge [10]. Few prevention programs in this setting are aimed at older adults [6].

Interest in HIV and aging is mounting as evidenced by the increasing body of literature focused on aging, the emergence of meetings such as the 1st International Workshop on HIV and Aging held in Baltimore, MD, in 2010, and a growing number of advocacy activities such as the National HIV/AIDS and Aging Awareness Day, held annually in the USA since 2009. In turn, focus on behavioral and psychosocial issues associated with HIV and aging is building. Emlet [13] found that older adults were less likely to disclose their HIV serostatus to relatives, partners, mental health workers, neighbors, and church members than those aged 20–39 years. Negin et al. [10] found similar results in sub-Saharan Africa. One US-based study found that older adults were more likely than their younger counterparts to be adherent to their antiretroviral therapy (ART) regimens [14]. In contrast, others [15] have found that adherence to ART and other medications decreases as the number of chronic conditions increases among HIV-positive older adults.

Though strides have been made in treatment scale-up in sub-Saharan Africa, very little is known about older adults accessing HIV care and treatment in resource-limited settings. This paper examines whether sociodemographic, behavioral, and clinical characteristics of those aged 50+ differ from those aged 18–49 years. Being over the age of 50 was a predictor of self-reported adherence according to a previous analysis of the women in this cohort [16]. The current paper seeks to extend these findings by evaluating whether or not there was an association between age and adherence to ART or other HIV-related medications in the overall International Epidemiologic Databases to Evaluate AIDS (IeDEA) Central Africa region cohort.

## 2. Materials and Methods

The HIV-infected adults included in this analysis were receiving care at 10 HIV care and treatment facilities contributing data to the IeDEA Central Africa region database. The National Institute of Allergy and Infectious Diseases funded the IeDEA initiative to establish regional centers for the collection and harmonization of HIV-related data. This international research consortium has enabled researchers in participating regions to better describe regional trends as well as address unique and evolving research questions in HIV/AIDS currently unanswerable by single cohorts. The Central Africa region database includes data from existing healthcare facilities in the Democratic Republic of the Congo (DRC), where data collection began in 2007, and Cameroon and Burundi, where data collection began in 2008. Approval for this research was granted by the Institutional Review Board (IRB) at the Kinshasa School of Public Health in DRC and RTI International, as well as the national ethics committees in Burundi and Cameroon.

Sites providing data to the IeDEA Central Africa database were a combination of public and private hospital and ambulatory care units of varying size ranging from three patient beds at one clinic in DRC to 300 beds at the largest hospital in Cameroon. Participating sites served predominantly urban populations and offered primary care in DRC and tertiary care in Burundi and Cameroon. All participating sites recommended and provided routine HIV testing for participants' relatives, sex partners, and household members and had some level of linkage to programs providing prevention of maternal to child transmission (PMTCT) services. Participating sites in Cameroon were the first within the IeDEA Central Africa region to offer free ART for adults in 2000, while participating sites in DRC started in 2005, followed by the Burundi site in 2006.

All of the clinic sites contributing data for this analysis provided individual adherence counseling for patients. Many sites also offered group counseling on medication adherence. Frequency of counseling ranged from site to site, with some programs only providing adherence counseling in the event of virologic failure, while others provided counseling at initiation of therapy, and at follow-up clinic visits every one to three months.

Some sites were also able to provide other types of ART adherence support. Many of the sites in the DRC used follow-up appointments to assess adherence, and some distributed tools such as written or illustrated instructions on when to take each medication and, to a lesser extent, calendars, alarm clocks, watches, or pagers to be used as reminders. Other sites used teaching techniques such as quizzes on how and when to take each medicine as a method of reinforcing the information learned in the counseling. At the Cameroon and Burundi sites, the medical teams also incorporated a pharmacist into multidisciplinary teams of providers and some sites had videos with instructions on adherence for patients to view.

All patient-level adherence data were self-reported and assessed at each individual's last visit prior to this analysis. All adults, regardless of whether they were on ARVs, were asked whether they had missed taking their medication more than two consecutive days in the last month. For those not on ARVs, missed medications included, most commonly, cotrimoxazole prophylaxis and, to a lesser extent, tuberculosis (TB) prophylaxis and TB treatment. Length of time on ARVs was calculated by determining the length of time between the ARV start date and the last follow-up visit prior to this analysis and was coded as not on ARVs, <6 months, 6–24 months, and >24 months. Those on ART were followed every one to three months and those not on ART were followed every six months, unless there was a clinical event for which they needed to return to the clinic for evaluation and/or care. All patient-level data used in this analysis were collected during a face-to-face interview with a clinic doctor or nurse.

## 3. Statistical Analysis

All analyses were performed using SAS 9.1 for Windows [17]. We examined baseline sociodemographic, behavioral, and clinical characteristics of those aged 50+ with those aged 18–49 years using chi-square tests to determine if distributions between the two groups differed. We evaluated differences between countries using chi-square tests to determine if distributions between DRC, Cameroon, and Burundi differed. We also examined whether age was associated with self-reported medication adherence. We defined nonadherence as missed doses (of ART or other HIV-related medications) for two or more consecutive days in the past 30 days. Logistic regression was used to determine if age was associated with medication adherence while controlling for variables such as country, marital status, gender, employment status, heavy drinking, education, clinical stage at enrolment into the IeDEA database, and length of time on ARVs. Included in the model were sociodemographic and clinical characteristics that we hypothesized a priori might affect adherence based on reported associations in the literature in the context of sub-Saharan Africa [18–20] while also considering completeness of data in the IeDEA Central Africa database.

## 4. Results

As of June 2011, there were 18,839 adults enrolled in HIV care in the IeDEA Central Africa region database and 2,819 (15%) were 50 years old or older (Table 1). The majority of adults (*N* = 10,647) were from DRC, 5,835 were from Cameroon, and 2,357 were from Burundi. Of adults aged 50+, the mean age in both DRC and Cameroon was 55 years (median 54 years) and 56 years in Burundi (median 55 years). Those aged 50+ were more evenly distributed between women and men (56% versus 44%, resp.) as compared to those aged 18–49 (71% versus 29%, resp.) (*P* < 0.05). Approximately 20% of both groups reported heavy drinking, defined as three or more alcoholic drinks per day on average.

Adults were asked about their marital status, whether they had any casual sex partners in the last 6 months (defined as an occasional sex partner in addition to the respondent's regular partner), whether they had a sex partner (regular or casual) that recently died, and whether they used condoms with their regular partner. One-quarter of those aged 18–49 reported being single, compared to only 5% of older adults (*P* < 0.05). Seventeen percent of adults 18–49 indicated they had a casual sex partner within the last 6 months as compared to 8% of those 50+ (*P* < 0.05). About half (42%) of adults aged 50+ reported having a sex partner that recently died as compared to 27% of those aged 18–49 years (*P* < 0.05). A higher proportion of those aged 18–49 reported using condoms with their regular partner (19%) as compared to those aged 50+ (11%) (*P* < 0.05).

We compared HIV serostatus disclosure of those aged 18–49 and 50+ at enrollment into the IeDEA Central Africa database. A higher proportion of those aged 18–49 as compared to those 50+ had shared their HIV test results with their partner or spouse (33% versus 27%, resp.) (*P* < 0.05). The majority had shared their results with a family member (57% for both groups), while few had shared their results with a friend (6% versus 4%, resp.), health worker (6% for both groups), or someone living in the home (2% for both groups). Few were referred for disclosure counseling at the baseline visit (3% versus 2%, resp.).

To examine whether older adults were living with fewer amenities than their younger counterparts, we reviewed four variables addressing socioeconomic status: education level, paid profession, access to electricity in the home, and running water in the home. Older adults were more likely to report no formal education than their younger counterparts (14% and 7%, resp.) (*P* < 0.05); however, there were no differences between the two age groups for having a paid profession (42% of both groups), electricity (approximately 78% of both groups) and running water (approximately 61% of both groups) in the home.

We compared the health status of those aged 18–49 and 50+ at enrollment into the IeDEA Central Africa database. The majority of both groups entered HIV care through voluntary counseling and testing (56% and 55%, resp.). The majority of both groups (64% and 65%, resp.) had moderate-to-severe HIV disease progression classified as WHO clinical stage 3 or 4 at enrollment into the IeDEA database. Of the 7,858 adults with CD4 counts available at enrolment into the IeDEA database, a higher proportion of those aged 18–49 years had CD4 cells counts less than 200 cells/mm^3^ (44%) as compared to 37% of adults 50+ (*P* < 0.05). A higher proportion of those aged 50+ (8%) had a history of hypertension as compared to those aged 18–49 years (3%) (*P* < 0.05) while few had a history of diabetes (3% versus 1%, resp.). About 20% of both groups had a history of tuberculosis.

Recognizing the diversity of the countries included in the IeDEA Central Africa region, we examined the sociodemographic, behavioral, and clinical characteristics of the 18,839 HIV+ adults in the database by country (Table 1). A higher percentage of adults in the Cameroon sites, (35%) were single as compared to adults in the DRC and Burundi sites (17% for both) (*P* < 0.05). Few adults in the DRC and Cameroon sites (4% for both) reported having no formal education as compared to 37% of adults in Burundi (*P* < 0.05). A higher percentage of adults in the Cameroon sites, reported having a paid profession (51%), electricity (93%) and running water (68%) in the home as compared to those in DRC and Burundi (*P* < 0.05).

Table 2 presents the results of the logistic regression model used to determine if age was associated with medication adherence while controlling for variables such as country, marital status, gender, employment status, heavy drinking, education, clinical stage at enrolment into the IeDEA database, and length of time on ARVs. Those aged 50+ were more likely to be adherent to their medications than those aged 18–49 (*P* < 0.001). Older adults had 1.59 times the odds of being adherent to their medications as compared to their younger counterparts. In terms of other predictors of adherence, adults who were not heavy drinkers had 1.40 times the odds of being adherent as compared to those who reported drinking three or more alcoholic beverages per day. Those who were not taking ARVs had 2.05 times the odds of being adherent to other medications (i.e., cotrimoxazole prophylaxis) as compared to those on ARVs for less than 6 months. Adults from the Burundi site had 2.23 times the odds of being adherent to their medications as compared to those from the DRC sites (*P* < 0.001). Adults from the Cameroon sites had 1.98 times the odds of being adherent to their medications as compared to those from the DRC sites (*P* < 0.001).

## 5. Discussion

Fifteen percent of this large cohort of HIV-infected adults were 50 years old or older. Though older adults were more likely to report no formal education than their younger counterparts, they did not seem to be living with fewer amenities as per the socioeconomic variables examined in this study: having a paid profession, access to electricity, and running water in the home. We found that older adults were more likely to be adherent to their medications than their younger counterparts. In terms of other predictors of adherence, we found that adults who were not heavy drinkers reported better adherence as compared to those who reported drinking three or more alcoholic beverages per day.

Older adults have been found to be more adherent to HIV medications, including ART, than their younger counterparts, which may improve their survival and response to treatment [14]. Though we found adults aged 50+ years to be more adherent to their medications than those aged 18–49, further inquiry is needed to determine if the older adults in this cohort, in turn, experience an improved response to ART and survival.

Alcohol abuse has been found to negatively affect adherence in sub-Saharan Africa (see Mills et al. [19] for a review) and our results provide further support. We found that adults who were not heavy drinkers reported better adherence as compared to those who reported drinking three or more alcoholic beverages per day. A similar proportion of older and younger adults reported heavy drinking (23% and 21%, resp.). However, it is important to note that those aged 50+ were more evenly distributed between women and men as compared to those aged 18–49, and, in this cohort, men tended to report alcohol use more frequently than women. Our results suggest that those reporting heavy alcohol use may benefit from additional adherence counseling. Those who were not taking ARVs reported better adherence to other medications (i.e., cotrimoxazole prophylaxis) as compared to those on ARVs for less than 6 months, supporting the notion that additional adherence counseling for new ART users may be beneficial.

Older adults in sub-Saharan Africa have been found to be less likely to discuss HIV prevention with their partner as compared to their younger counterparts [10]. The results of the current study echo these findings. Disclosure of HIV test results with partners/spouse as well as condom use with regular partner was higher among those aged 18–49 as compared to those aged 50+. Further, HIV programming in sub-Saharan Africa is generally targeted towards younger adults, not those over 50 [3, 21]. Older adults as compared to their younger counterparts have been found to be less likely to have been tested for HIV [10] and experience delays in diagnosis and treatment [8, 9], as clinicians may not routinely screen older adults for HIV or recognize their signs and symptoms as those of HIV [11]. In the current study, the majority of both age groups had moderate-to-severe HIV disease progression classified as WHO clinical stage 3 or 4 at enrollment into the IeDEA database, which corresponded to entry into HIV care for many adults.

Older adults are at increased risk for HIV infection due to biological and social factors [5, 6]. For women, in particular, age is associated with thinning of vaginal membranes and reduced vaginal lubrication, which can lead to tearing during sexual intercourse [5]. Social factors may place older adults at risk for HIV, such as divorce or death of a spouse, which may lead to new sexual partners and thus risk of exposure [5]. In sub-Saharan Africa, cultural practices, such as wife inheritance, may also place women at risk of contracting HIV in the event of the death of a spouse [6]. In the current study, a greater proportion of older adults as compared to those aged 18–49 were widowed (40% versus 18%, resp.) or divorced (11% versus 9%, resp.).

## 6. Limitations

Our results should be considered in light of several study limitations. The adherence data for this study were self-reported and collected during face-to-face interviews with a clinic doctor or nurse, which can lead to social desirability bias and, in turn, inflated adherence estimations [22]. Our baseline data were derived at enrollment into the IeDEA Central Africa database. For many adults, this also corresponded to enrollment into HIV care. Though we were able to determine when ART was started, we were not able to assess how long the patient had been in HIV care before enrolling into the IeDEA database.

The data presented in this paper provide a snapshot of patient characteristics from a broad range of private and public hospitals and ambulatory care units of varying size and capacity. However, the data are not nationally representative as they were not derived from randomly selected HIV care facilities. Though describing regional trends was an objective of the larger study, exploring differences between age groups was not an original goal. Collecting additional data on psychosocial variables, such as social support, depression, stigma, and quality of life, would have been insightful for examining potential differences between older adults and their younger counterparts.

## 7. Conclusions

This is the first study to examine whether there are differences between older adults and their younger counterparts accessing HIV care and treatment in the central Africa region. These results are noteworthy as they provide insight into the sociodemographic, behavioral, and clinical characteristics of HIV-infected older adults in this region. Though we found older adults were more likely to be adherent to their medications than their younger counterparts, further inquiry is needed to better understand factors affecting ART adherence, response to treatment, and survival of older adults receiving HIV care in sub-Saharan Africa. We found that heaving drinking negatively affected medication adherence, which suggests that those reporting heavy alcohol use may benefit from additional adherence counseling.

## Figures and Tables

**Table 1:** Characteristics of 18,839^a^ HIV+ adults in IeDEA Central Africa database presented by age (18–49 years, 50+ years) and by country (DRC, Cameroon, Burundi).

Characteristic	18–49 years *N* (%)	50+ years *N* (%)	DRC *N* (%)	Cameroon *N* (%)	Burundi *N* (%)
Sex*	*15,979*	*2,812*	*10,605*	*5,834*	*2,352*
Male	4,573 (28.6)	1,243 (44.2)	3,348 (31.6)	1,749 (30.0)	719 (30.6)
Female	11,406 (71.4)	1,569 (55.8)	7,257 (68.4)	4,085 (70.0)	1,633 (69.4)

*Marital status* ^∗*∧*^	*15,662*	*2,781*	*10,252*	*5,835*	*2,356*
Single	4,002 (25.6)	138 (5.0)	1,694 (16.5)	2,053 (35.2)	393 (16.7)
Divorced	1,345 (8.6)	311 (11.2)	1,110 (10.8)	316 (5.4)	230 (9.8)
Widowed	2,807 (17.9)	1,120 (40.3)	2,469 (24.1)	916 (15.7)	542 (23.0)
Living together-not married	1,622 (10.4)	74 (2.7)	732 (7.1)	624 (10.7)	340 (14.4)
Monogamous marriage	5,407 (34.5)	1,009 (36.3)	3,841 (37.5)	1,751 (30.0)	824 (35.0)
Polygamous marriage	479 (3.1)	129 (4.6)	406 (4.0)	175 (3.0)	27 (1.1)

*Education^∗*∧*^*	*15,999*	*2,808*	*10,616*	*5,835*	*2,356*
None	1,110 (6.9)	385 (13.7)	371 (3.5)	253 (4.3)	871 (37.0)
Primary school	6,915 (43.2)	959 (34.2)	4,450 (41.9)	3,006 (51.5)	418 (17.7)
Secondary school	6,260 (39.1)	1,134 (40.4)	4,447 (41.9)	1,935 (33.2)	1,012 (43.0)
University	1,714 (10.7)	330 (11.8)	1,348 (12.7)	641 (11.0)	55 (2.3)

*Socioeconomic indicators*					
Paid profession*^*∧*^*	6,727 (42.1)	1,187 (42.2)	4,127 (38.9)	2,984 (51.1)	803 (34.1)
Electricity in the home^*∧*^	12,623 (78.9)	2,204 (78.3)	8,419 (79.3)	5,417 (92.8)	991 (42.1)
Running water in the home*^*∧*^*	9,686 (60.5)	1,733 (61.6)	6,568 (61.8)	3,967 (68.0)	884 (37.5)

*Entry into HIV care^∗*∧*^*	*15,918*	*2,796*	*10,522*	*5,835*	*2,357*
PMTCT	381 (2.4)	7 (0.3)	69 (0.7)	246 (4.2)	73 (3.1)
TB clinic	495 (3.1)	65 (2.3)	262 (2.5)	277 (4.7)	21 (0.9)
STI clinic	330 (2.1)	75 (2.7)	59 (0.6)	341 (5.8)	5 (0.2)
VCT	8,902 (55.9)	1,523 (54.5)	6,164 (58.6)	2,343 (40.2)	1,918 (81.4)
No previous care	4,032 (25.3)	778 (27.8)	3,292 (31.3)	1,447 (24.8)	71 (3.0)
Other	1,778 (11.2)	348 (12.4)	676 (6.4)	1,181 (20.2)	269 (11.4)

*Clinical stage (WHO) at enrollment into IeDEA database^∗*∧*^*	*15,900*	*2,800*	*10,530*	*5,819*	*2,351*
1	2,387 (15.0)	303 (10.8)	989 (9.4)	915 (15.7)	787 (33.5)
2	3,348 (21.1)	658 (23.5)	2,469 (23.4)	1,136 (19.5)	401 (17.1)
3	8,603 (54.1)	1,588 (56.7)	6,443 (61.2)	2,911 (50.0)	836 (35.6)
4	1,562 (9.8)	251 (9.0)	629 (6.0)	857 (14.7)	327 (13.9)

*CD4 count at enrollment into IeDEA database^∗*∧*^*	*6,737*	*1,121*	*1,723*	*4,862*	*1,273*
<200	2,982 (44.3)	413 (36.8)	706 (41.0)	2,310 (47.5)	379 (29.8)
200–350	1,921 (28.5)	353 (31.5)	502 (29.1)	1,405 (28.9)	367 (28.8)
>350	1,834 (27.2)	355 (31.7)	515 (29.9)	1,147 (23.6)	527 (41.4)

*Length of time on ARVs^∗*∧*^*	*8,912*	*1,644*	*5,525*	*3,737*	*1,304*
<6 months	1,467 (16.5)	239 (14.5)	1,324 (24.0)	297 (7.9)	85 (6.5)
6–24 months	2,404 (27.0)	388 (23.6)	1,436 (26.0)	1,007 (26.9)	346 (26.5)
>24 months	5,041 (56.6)	1,017 (61.9)	2,765 (50.0)	2,433 (65.1)	873 (66.9)

*Comorbidity history*					
History of TB^*∧*^	3,237 (20.2)	538 (19.1)	2,553 (24.1)	669 (11.5)	553 (23.5)
History of diabetes^∗*∧*^	213 (1.3)	71 (2.5)	194 (1.8)	68 (1.2)	22 (0.9)
History of high blood pressure^∗*∧*^	501 (3.1)	234 (8.3)	492 (4.6)	163 (2.8)	80 (3.4)

*Has had a casual sex partner in last 6 months^∗*∧*^*	2,673 (16.7)	232 (8.2)	1,082 (10.2)	1,569 (26.9)	254 (10.8)

*Sex partner (regular or casual) who has recently died^∗*∧*^*	4,360 (27.2)	1,188 (42.1)	2,703 (25.4)	1,955 (33.5)	890 (37.8)

*Condom use with regular partner^∗*∧*^*	2,968 (18.5)	311 (11.0)	985 (9.3)	1,648 (28.2)	646 (27.4)

*Heavy drinking* (on average 3+ drinks/day)^∗*∧*^	3,369 (21.1)	636 (22.7)	2,207 (20.9)	1,389 (23.8)	409 (17.4)

*HIV test result disclosure*					
Partner/spouse^∗*∧*^	5,255 (32.8)	771 (27.4)	2,755 (25.9)	2,093 (35.9)	1,178 (50.0)
Friend^∗*∧*^	908 (5.7)	113 (4.0)	277 (2.6)	406 (7.0)	338 (14.3)
Family member^*∧*^	9,116 (56.9)	1,618 (57.4)	5,693 (53.5)	3,959 (67.8)	1,082 (45.9)
Health worker^*∧*^	881 (5.5)	169 (6.0)	254 (2.4)	776 (13.3)	20 (0.8)
Someone living in the home^*∧*^	311 (1.9)	68 (2.4)	308 (2.9)	39 (0.7)	32 (1.4)
Referral made for disclosure counseling	528 (3.3)	61 (2.2)	115 (1.1)	471 (8.1)	3 (0.1)

^a^Variables do not add to total number of adults in the database (18,839) due to missing data.

*Significant differences found between adults 18–49 years and adults 50+ years distributions (*α* = 0.05).

^*∧*^Significant differences found between DRC, Cameroon, and Burundi distributions (*α* = 0.05).

**Table 2:** Adjusted^1^ odds ratios and 95% confidence intervals for the association of medication adherence^2^ and age.

Variable	*Medication adherence * Odds ratio	95% confidence interval	*P* value
*Main effect variable*			
Age			
18–49 years	*Ref*		<0.001
50+ years	1.59*	(1.21, 2.09)	

*Covariates*			
Country			
DRC	*Ref*		<0.001
Burundi	2.23*	(1.57, 3.15)	
Cameroon	1.98*	(1.60, 2.46)	

Marital status			
Single	*Ref*		0.848
Divorced	1.05	(0.76, 1.46)	
Widowed	1.11	(0.85, 1.44)	
Married/living together (includes monogamous and polygamous marriage)	1.09	(0.88, 1.36)	

Sex			
Female	*Ref*		0.569
Male	0.95	(0.78, 1.15)	

Employment status			
No paid employment	*Ref*		0.813
Paid employment	1.02	(0.86, 1.22)	

Heavy drinking			
Yes	*Ref*		<0.001
No	1.40*	(1.15, 1.70)	

Education level			
Secondary/University	*Ref*		0.216
Primary	0.94	(0.79, 1.12)	
No formal education	0.72	(0.50, 1.05)	

Clinical stage (WHO) at enrollment into IeDEA database			
I/II	*Ref*		0.774
III/IV	0.97	(0.82, 1.17)	

Length of time on ARVs			
<6 months	*Ref*		<0.001
6–24 months	0.69*	(0.53, 0.89)	
>24 months	1.12	(0.87, 1.45)	
Not on ARVs	2.05*	(1.51, 2.78)	

*P < 0.001.

^1^Adjusted for country, marital status, gender, employment status, heavy drinking, education, clinical stage at enrollment into IeDEA database, and length of time on ARVs.

^2^Nonadherence was defined as missed doses of ART or other HIV-related medications (most commonly cotrimoxazole prophylaxis and, to a lesser extent, TB prophylaxis and TB treatment) for two or more consecutive days in the past 30 days.

## References

[2] Centers for Disease Control and Prevention (2008). HIV/AIDS among persons aged 50 and older: CDC HIV/AIDS facts. http://www.cdc.gov/hiv/topics/over50/resources/factsheets/pdf/over50.pdf.

[3] Mills EJ, Rammohan A, Awofeso N (2011). Ageing faster with AIDS in Africa. *The Lancet*.

[4] Centers for Disease Control and Prevention Behavioral risk factor surveillance system survey data 2000.

[5] Vance DE, Childs G, Moneyham L, Mckie-Bell P (2009). Successful aging with HIV: a brief overview for nursing. *Journal of Gerontological Nursing*.

[6] Negin J, Cumming RG (2010). HIV infection in older adults in sub-Saharan Africa: extrapolating prevalence from existing data. *Bulletin of the World Health Organization*.

[7] Adler WH, Baskar PV, Chrest FJ, Dorsey-Cooper B, Winchurch RA, Nagel JE (1997). HIV infection and aging: mechanisms to explain the accelerated rate of progression in the older patient. *Mechanisms of Ageing and Development*.

[8] Luther VP, Wilkin AM (2007). HIV infection in older adults. *Clinics in Geriatric Medicine*.

[9] Uphold CR, Maruenda J, Yarandi HN, Sleasman JW, Bender BS (2004). HIV and older adults: clinical outcomes in the era of HAART. *Journal of Gerontological Nursing*.

[10] Negin J, Nemser B, Cumming R, Lelerai E, ben Amor Y, Pronyk P HIV attitudes, awareness and testing among older adults in Africa.

[11] Oyieng'o DO, Bradley J (2010). HIV in the older adult. *Medicine and Health, Rhode Island*.

[12] Orel NA, Wright JM, Wagner J (2004). Scarcity of HIV/AIDS risk-reduction materials targeting the needs of older adults among state departments of public health. *Gerontologist*.

[13] Emlet CA (2006). A comparison of HIV stigma and disclosure patterns between older and younger adults living with HIV/AIDS. *AIDS Patient Care and STDs*.

[14] Silverberg MJ, Leyden W, Horberg MA, DeLorenze GN, Klein D, Quesenberry CP (2007). Older age and the response to and tolerability of antiretroviral therapy. *Archives of Internal Medicine*.

[15] Catz S, Balderson B, BlueSpruce J, Mahoney C, Harrison R, Grothaus L Chronic disease burden association with medication adherence and quality of life in an older HIV population.

[16] Freeman A, Newman J, Hemingway-Foday J Comparison of HIV-positive women with children and without children accessing HIV care and treatment in the IeDEA Central Africa cohort.

[17] The SAS Institute Inc. The SAS System for Windows (Version 9.1.3).

[18] Carlucci JG, Kamanga A, Sheneberger R (2008). Predictors of adherence to antiretroviral therapy in rural Zambia. *Journal of Acquired Immune Deficiency Syndromes*.

[19] Mills EJ, Nachega JB, Buchan I (2006). Adherence to antiretroviral therapy in sub-Saharan Africa and North America: a meta-analysis. *Journal of the American Medical Association*.

[20] Tiyou A, Belachew T, Alemseged F, Biadgilign S (2010). Predictors of adherence to antiretroviral therapy among people living with HIV/AIDS in resource-limited setting of southwest Ethiopia. *AIDS Research and Therapy*.

[21] Kimokoti RW, Hamer DH (2008). Nutrition, health, and aging in sub-Saharan Africa. *Nutrition Reviews*.

[22] Berg KM, Arnsten JH (2006). Practical and conceptual challenges in measuring antiretroviral adherence. *Journal of Acquired Immune Deficiency Syndromes*.

